# Photothermal Properties of Solid-Supported Gold Nanorods

**DOI:** 10.1021/acs.nanolett.4c03472

**Published:** 2024-09-30

**Authors:** Maja Uusitalo, Michal Strach, Gustav Eriksson, Tetiana Dmytrenko, John Andersson, Andreas Dahlin, Mats Hulander, Martin Andersson

**Affiliations:** † Department of Chemistry and Chemical Engineering, Chalmers University of Technology, SE-412 96 Gothenburg, Sweden; ‡ Chalmers Materials Analysis Laboratory, Chalmers University of Technology, SE-412 96 Gothenburg, Sweden

**Keywords:** gold nanorods, near-infrared, thermoplasmonics, X-ray diffraction, thermal expansion

## Abstract

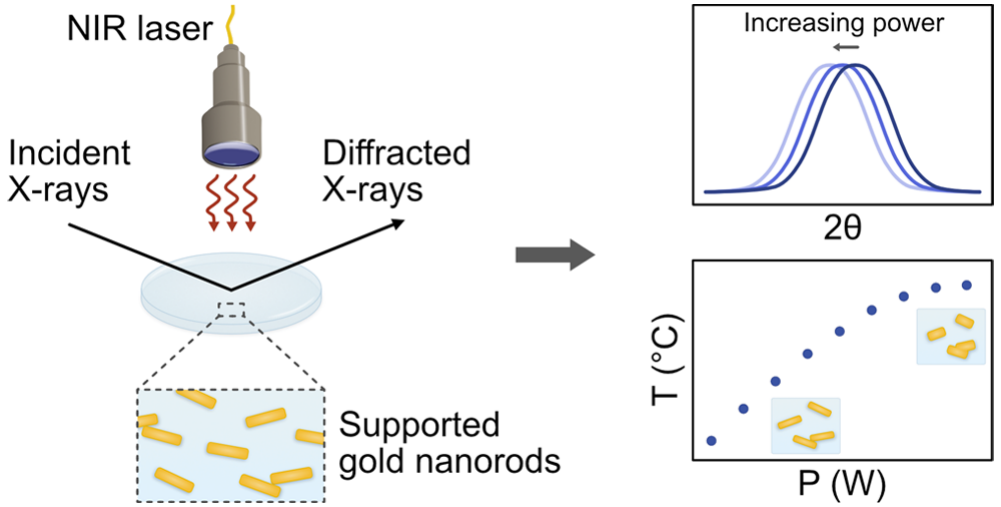

Gold nanoparticles possess unique photothermal properties and have gained considerable interest in biomedical research, particularly for photothermal therapy (PTT). This study focuses on evaluating the photothermal properties of gold nanorods (AuNRs) supported on glass substrates upon excitation with near-infrared (NIR) light. Two aspect ratios of AuNRs were electrostatically immobilized onto glass with controlled coverage. *In situ* X-ray diffraction (XRD) was performed to evaluate the photothermal behavior and morphological changes of the supported AuNRs during NIR laser irradiation. The XRD data sets were corroborated with scanning electron microscopy and Vis-NIR spectroscopy characterization. XRD revealed a linear temperature increase with laser power, aligning with theoretical predictions, and a slope dependent on the AuNR coverage, until the onset of morphology transformations around 120 °C. This study provides valuable insights into the photothermal properties of supported AuNRs, crucial for their application in PTT.

The photothermal properties of gold nanoparticles are of great interest in biomedical research, with potential uses for a range of diagnostic and therapeutic purposes. One promising application within biomedicine is photothermal therapy (PTT), wherein the heat released from the gold nanoparticles upon excitation with resonant light is used to treat a medical condition.^1,2^ The gold nanoparticles’ tunable optical properties and large absorption cross section, together with their localized heat emission, allow them to act as effective nanoscale heat sources. By tuning the size and shape of the gold nanoparticles during synthesis, the resonance frequency can be optimized to match the so-called biological window in the near-infrared (NIR) region where optimal tissue penetration is observed, enabling extracorporeal excitation and heat generation.^3^

Extensive literature on the use of gold nanoparticles of different shapes and sizes for photothermal cancer therapy exists, and several review articles cover the topic.^1,4^ The concept has also been investigated as an alternative to conventional antibacterial agents in the treatment of bacterial infections. As such, they have been used both free in suspension^5−7^ and immobilized on substrates.^8−10^ For these applications, which rely on local hyperthermia to damage a targeted group of cells, it is of importance to understand and control the photothermal properties of the gold nanoparticles. However, because the heat generated from gold nanoparticles upon excitation with resonant light is spatially confined to the nanoscale, determining the temperature of these photothermal systems is challenging.

A range of methodologies has been developed for experimental assessment of the local temperature increase caused by irradiation of plasmonic nanoparticles and have been reviewed previously.^11^ Many of the methods assess some change in property in the medium surrounding the gold nanoparticles to evaluate the local temperature, for example by using fluorescent probe molecules^12,13^ or upconverting nanoparticles,^14^ by monitoring changes in viscosity or refractive index,^15,16^ or by using the phase transition of lipid bilayers.^17,18^ Several of the developed methodologies have demonstrated good accuracy and cover a relatively wide temperature span. However, as most rely on monitoring changes in the properties of the surrounding medium, they are indirect and only determine the temperature in proximity of the nanoparticles. Furthermore, the techniques that rely on optical detection have limitations in spatial resolution, due to the diffraction limit of visible light.^11^

In contrast, a direct approach monitors changes in the nanoparticles’ intrinsic properties, specifically utilizing the crystal lattice expansion resulting from heating of the gold nanoparticles to determine their temperature. By using X-ray diffraction (XRD) to monitor shifts and shape changes in the Bragg peaks of the crystalline gold nanoparticles as they are exposed to resonant light, it is possible to determine not only their exact temperature, but also to monitor structural changes in the particles. Previous work at synchrotron facilities on spherical gold nanoparticles in water^19^ and on silicon substrates^20^ using pulsed laser excitation has shown the effectiveness of this approach, studying for instance the temperature increase as a function of laser power, premelting and recrystallization of the particles, and heat-transfer to the support material. Extending this methodology to evaluating more biomedically relevant anisotropic gold nanoparticles excited by NIR light has the potential to provide valuable insights for their use in PTT applications.

In the present work, we have evaluated the photothermal properties of gold nanorods (AuNRs) supported on glass substrates, a model system which has previously been employed in PTT applications to demonstrate the antibacterial activity of supported AuNRs and NIR light.^10^ Both the effect of the nanoparticle shape, a parameter that is known to influence their properties, and the collective heating were investigated. For this purpose, two populations of AuNRs with different aspect ratios were synthesized and electrostatically immobilized onto glass substrates. Additionally, collective heating effects were investigated by adjusting the surface coverage of AuNRs on the support. *In situ* XRD studies were performed to evaluate the temperature and morphological transformations of the supported AuNRs during furnace heating and NIR laser irradiation at different powers. The *in situ* data sets were corroborated with sscanning electron microscopy (SEM) and Vis-NIR spectroscopy characterization, as well as compared to theoretical predictions of the heating of plasmonic nanoparticle arrays.

The two different populations of gold nanorods were synthesized via wet-chemical, seed-mediated procedures, resulting in average aspect ratios of 3.9 (AuNR 3.9) and 4.4 (AuNR 4.4). The dimensions of the AuNRs were determined from transmission electron microscopy (TEM) micrographs to be 67 ± 7 × 18 ± 2 nm and 66 ± 10 × 15 ± 2 nm for AuNR 3.9 and AuNR 4.4, respectively. Details are available in Supporting Information (Table S.2 and Figure S.2). High-resolution TEM characterization revealed the fcc single-crystalline nature of both aspect ratios of AuNRs (Figure 1A,B). The lattice fringes observed have distances in good agreement with the (200) planes in gold (0.203 nm) parallel and perpendicular to the long axis of the nanorods.

**Figure 1:**
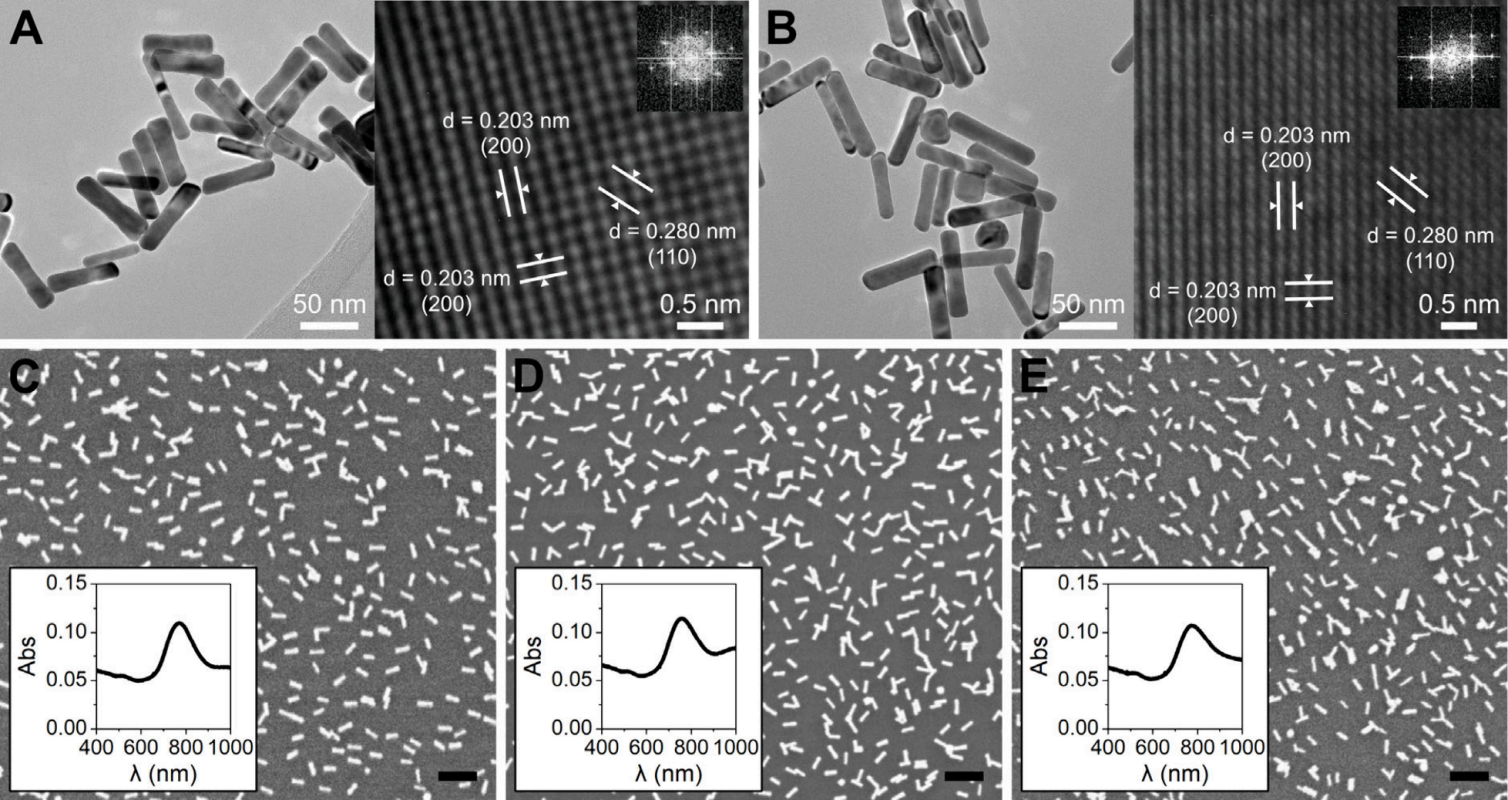
TEM characterization of (A) AuNR 3.9, and (B) AuNR 4.4. SEM micrographs and Vis-NIR absorption spectra of the AuNRs supported on glass for (C) AuNR 3.9 with 10.7 ± 2.0% surface coverage (AuNR 3.9 11%), (D) AuNR 3.9 with 13.4 ± 1.4% surface coverage (AuNR 3.9 13%), and (E) AuNR 4.4 with 11.0 ± 1.4% surface coverage (AuNR 4.4 11%). Scale bars in (C), (D) and (E) are 200 nm.

To prepare the samples for evaluating the photothermal properties, the AuNRs were immobilized onto glass substrates via electrostatic interaction. Three types of samples were used in the *in situ* XRD studies: (1) AuNR 3.9 with a surface coverage of 10.7 ± 2.0% (Figure 1C, AuNR 3.9 11%), (2) AuNR 3.9 with a surface coverage of 13.4 ± 1.4% (Figure 1D, AuNR 3.9 13%), and (3) AuNR 4.4 with a surface coverage of 11.0 ± 1.4% (Figure 1E, AuNR 4.4 11%). The three sample types were prepared to enable evaluating the influence of AuNR morphology and surface coverage on the photothermal properties.

From the absorption spectra in Figure 1C–E we observed that the nanorods retain their plasmonic properties once immobilized on the glass substrates, and that the longitudinal surface plasmon resonance (SPR) peak maxima overlap well with the 808 nm NIR laser used. The surface-immobilization procedure generated an even coverage of AuNRs on the glass, where the particles are randomly attached, mainly as individual AuNRs or in small clusters. To evaluate the degree of clustering in each sample type, we determined the fraction (in area%) of the surface coverage comprised by individual AuNRs. For AuNR 3.9 11%, individual AuNRs comprise 62% of the covered area, for AuNR 3.9 13%, individual AuNRs comprise 34%, and for AuNR 4.4 11%, individual AuNRs comprise 39%.

The strong orientation of the supported single crystal AuNRs is demonstrated by the absence of the (111) diffraction peak in the Bragg–Brentano scans of a flat mounted sample, where only a broad (200) peak was observed (XRD patterns of untreated AuNR 3.9 11% and AuNR 4.4 11% in Supporting Information, Figure S.3). In some diffractograms we observed a faint (111) peak, hardly distinguishable from the significant background from the glass substrate. Further texture analysis experiments using a Eulerian cradle stage were performed to determine the orientation of the crystallites in more detail. From the texture analysis (Figure S.4), the majority of AuNRs were found to be single crystals with the (200) plane parallel to the long axis, which is also evident in the TEM micrographs (Figure 1A,B), as well as to be oriented with the long axis toward the support. This orientation is also confirmed in the SEM micrographs (Figure 1C–E).

We performed a series of *in situ* XRD heating experiments on the three sample types (Figure 1C–E), using conventional furnace heating cells, as well as NIR heating under constant illumination using an 808 nm laser. By tracking the position and characteristics of the (200) peak, we followed the evolution of the structure and morphology of the supported AuNRs during heating. The main series of furnace heating experiments were performed on a D8 Advance diffractometer (Bruker) equipped with an XRK900 heating chamber (Anton-Paar). The NIR laser heating experiments were carried out on a D8 Discover (Bruker) with the laser placed within the enclosure of the instrument (illustrated in Figure 2).

**Figure 2:**
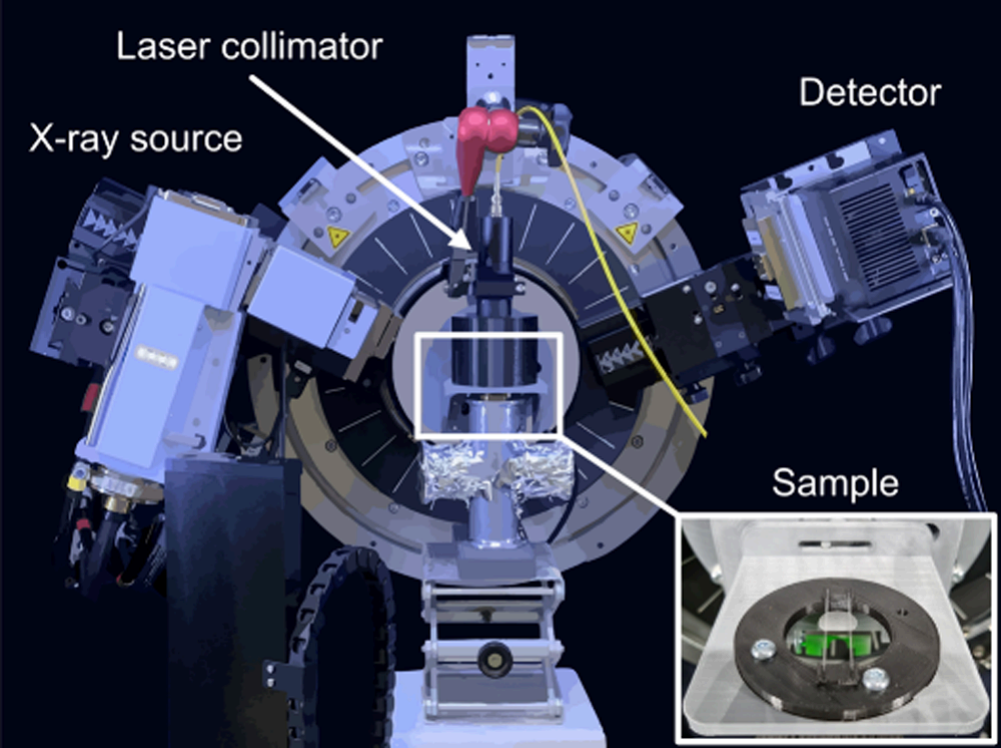
Schematic illustration of the setup used for the *in situ* XRD NIR laser experiments. The samples were placed on a support stage consisting of two glass capillaries (inserted picture), ensuring that the NIR beam did not heat any other elements than the sample. The NIR laser system’s collimator was placed 10 cm away from the sample at a 90° angle, illuminating the entire sample surface and ensuring that the flux was comparable between experiments.

Figure 3A shows the evolution of the (200) peak during the furnace heating experiments in the XRK900 cell for AuNR 4.4 11%. Based on the position of the (200) peak, we determined the lattice parameter as a function of the calibrated temperature for the AuNR 3.9 11% and AuNR 4.4 11% samples, shown in Figure 3B (raw data in Figure S.5). The *Bulk* lattice parameter was calculated using the thermal expansion coefficient for gold (eq 1).^21^ The expansion coefficients of the AuNRs appear to be like those reported in the literature for bulk gold in the considered temperature range, within the limits of instrumental resolution.
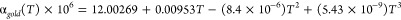
1

**Figure 3:**
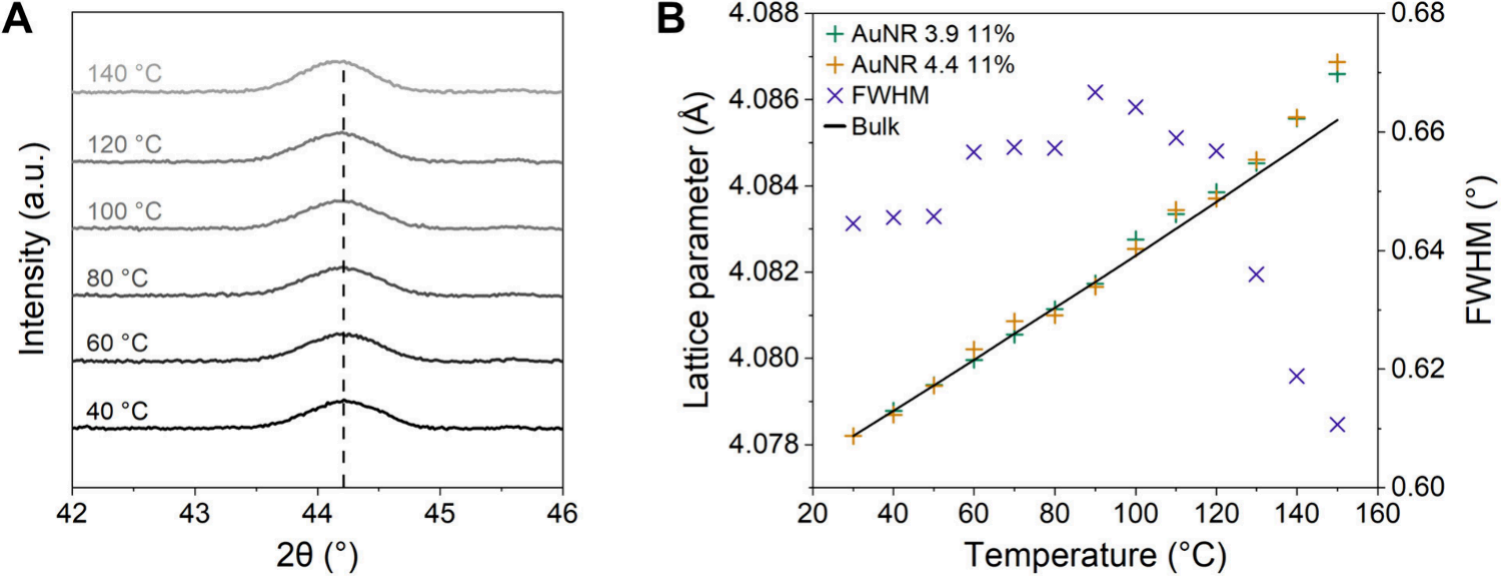
(A) The evolution of the (200) peak during the furnace heating for AuNR 4.4 11%. (B) Lattice parameter of the gold cubic structure for AuNR 3.9 11% and AuNR 4.4 11%, together with FWHM for AuNR 4.4 11%, plotted against temperature. The solid line represents the thermal expansion of bulk gold (eq 1). We attribute the sharp decrease in FWHM above 120 °C to changes in the morphology of the AuNRs.

In Figure 3B, we furthermore show the evolution of full width at half-maximum (FWHM) of the (200) peak for AuNR 4.4 11%, using it as a probe for morphological changes in the nanoparticles, wherein a decrease in FWHM marks the onset of morphology transformations. The evolution of the Au (200) diffraction peak during heating corresponds purely to thermal expansion of the AuNRs with no apparent morphological changes up to around 120 °C. At temperatures above 120 °C, morphological transformations, manifesting as irreversible modification of the peak’s shape, were observed. In another series of measurements using a Linkam heating cell, we noted that the evolution of peak shapes continues until at least 250 °C (Figure S.6).

Postexperiment characterization, shown for AuNR 3.9 11% in Figure 5, of the samples heated to 250 °C shows a decrease in aspect ratio of individual AuNRs and the coalescence of clusters of AuNRs into larger, more spherical structures, as well as pronounced changes in the Vis-NIR absorption characteristics. The complete characterization data is included in Supporting Information. Interestingly, the coalescence into clusters apparently occurs at a lower temperature compared to the complete transformation of individual nanorods into spheres. This is likely due to the complex surface of an AuNR cluster, which leads to an increased free energy.

The crystallite orientation of the AuNRs was not affected even after heating to 250 °C, which is demonstrated by the continued absence of the (111) peak in the diffraction pattern (Figure S.4). This observation suggests that the crystalline core of the nanoparticles was preserved and served as a nucleus for the reorganization of outer layers, as observed in a molecular dynamics study of similar systems.^22^

For further understanding of the *in situ* NIR laser experiments, we considered a theoretical model for the heating of plasmonic nanoparticle (NP) arrays. The temperature increase in the center of a 2D infinite NP array exposed to a circular irradiation spot can be estimated from^23^
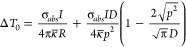
2

Here, σ_*abs*_ is the NP absorption cross section, *I* the laser irradiance, κ̅ the average thermal conductivity of the support and the medium, *R* the nanoparticle radius, *D* the diameter of the heated area, and *p* the periodicity in the case of a square lattice. eq 2 predicts a linear temperature increase with laser power, given that the absorption cross section and periodicity remain constant. Furthermore, it shows a dependence on the number of NPs per unit area, wherein a greater temperature increase is expected for a lower value of the periodicity.

For a perfect regular 2D array of plasmonic NPs, it is further possible to estimate the relative contribution of the self-temperature increase of a NP to the external temperature increase from the surrounding NPs in a dimensionless parameter, *ζ*_*2*_.^23^ For the systems studied here, estimating ζ_2_ revealed that collective heating effects were predicted to dominate (calculations in Supporting Information), with a homogeneous temperature increase throughout the entire AuNR array.

During the *in situ* XRD NIR laser experiments, the samples were exposed to continuous NIR light with varying power up to 24 W and we followed the thermal dilatation of the AuNRs, which resulted in a shift of the (200) peak position to lower angles (raw XRD data in Figure S.7). Based on a comparison of the measured lattice expansion for the supported AuNRs to literature values for bulk gold (Figure 3B), we established a relation between the laser power and temperature for AuNR 3.9 11%, AuNR 4.4 11% and AuNR 3.9 13%, shown in Figure 4A.

**Figure 4:**
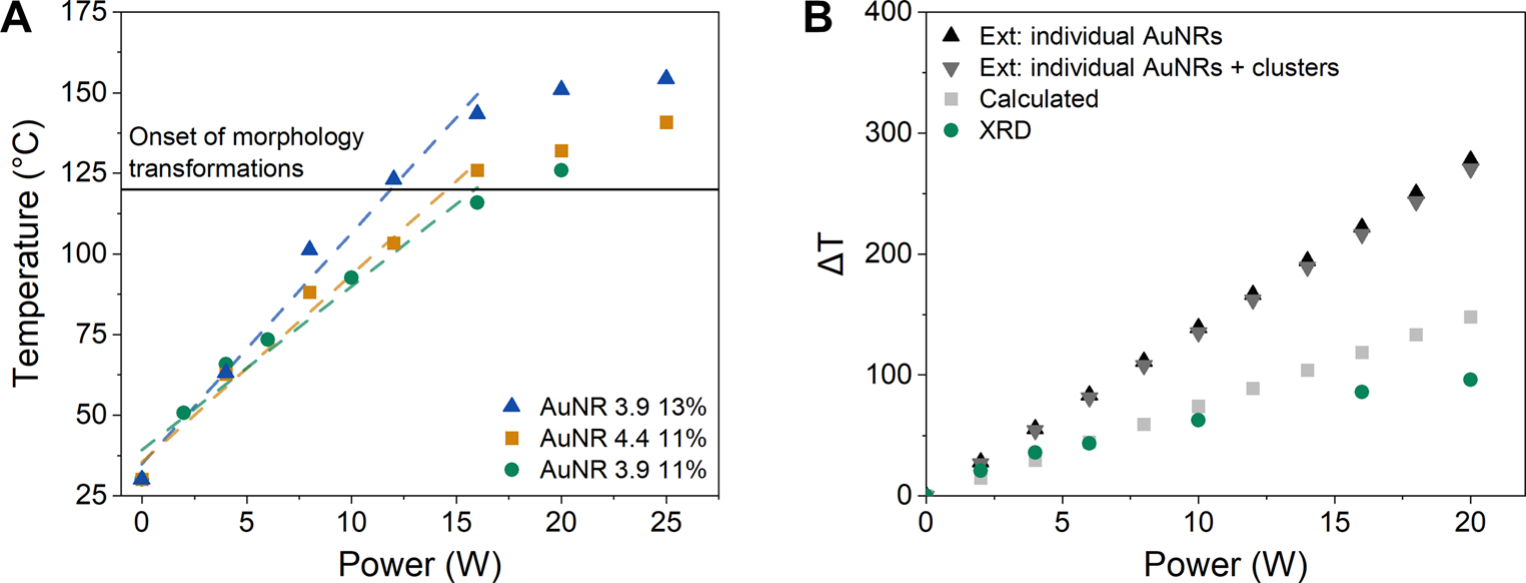
(A) Temperature as a function of laser power determined from comparing the measured lattice parameters with the thermal expansion of gold, for samples AuNR 3.9 11%, AuNR 4.4 11% and AuNR 3.9 13%. Dashed lines represent linear trends up to 120 °C. (B) Theoretically predicted (eq 2) and experimentally determined temperature increase as a function of laser power for AuNR 3.9 11%. The absorption cross sections used in the theoretical predictions were determined by calculations based on the AuNR dimensions (“Calculated”), or based on extinction spectroscopy measurements assuming either only individual AuNRs (“Ext: individual AuNRs”) or both individual and clustered AuNRs (“Ext: individual AuNRs + clusters”) contribute to the extinction at 808 nm.

From the *in situ* XRD studies (Figure 4A) we observed a linear temperature increase with laser power for the supported AuNRs, aligning with the theoretical model, until the onset of morphology transformations around 120 °C. In the linear temperature-power region, we expect no significant impact on the photothermal properties from a shift in the SPR peak induced by thermal expansion of the crystal lattice of the AuNRs. This was validated by monitoring the extinction peak position during heating of the samples up to 110 °C (Figure S.13) and corroborated with theoretical predictions (details in Supporting Information). The slope of the plots in Figure 4A increases with surface coverage, and upon increasing the average surface coverage of AuNR 3.9 from 10.7% to 13.4%, a corresponding 1.4-fold augmentation in the attained temperature at a given power level was observed.

The results in Figure 4A show that it is possible to detect the onset of nanoparticle morphology transformations during NIR-induced heating by monitoring the lattice thermal expansion with XRD. For the supported AuNRs the transformations begin around 120 °C, causing deviations from the linear temperature-power relationship, and they are accompanied by modifications of the optical properties, as clearly indicated in the Vis-NIR spectra of laser-heated samples (Figure 5, Figure S.11). As the aspect ratio of the AuNRs decreases, the longitudinal absorption peak blue-shifts; hence the absorption efficiency at 808 nm is lowered, in the end making it impossible to further heat the sample by increasing laser power.

**Figure 5:**
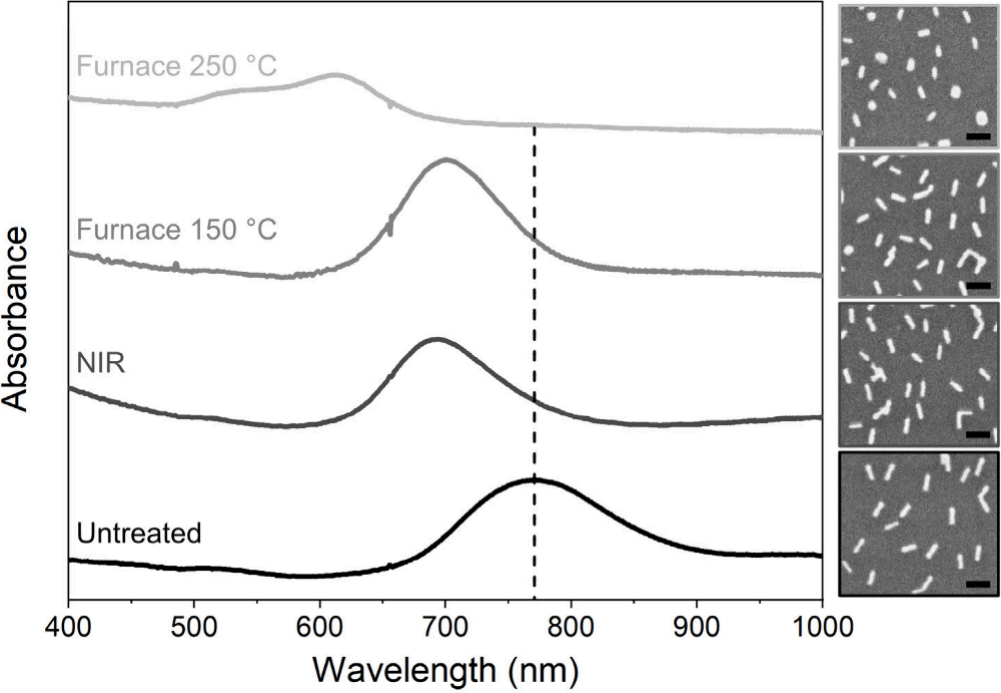
Postexperiment Vis-NIR spectroscopy and SEM characterization of AuNR 3.9 11%, showing the morphology transformations and the resulting absorption peak shifts induced by furnace heating or NIR laser irradiation. Scale bars in the SEM micrographs are 100 nm.

To compare the experimental findings with the theoretical model (eq 2), we determined the extinction and absorption cross sections of the AuNRs from (1) calculations based on the nanoparticle dimensions, and (2) extinction spectroscopy measurements (details in Supporting Information). From extinction spectroscopy, the cross sections were determined assuming that either only the individual AuNRs contribute to the extinction at 808 nm, or that both individual and clustered AuNRs contribute. In addition, the absorption cross sections calculated based on the nanoparticle dimensions, along with the surface coverage, were used to estimate the photothermal conversion efficiency of the samples (details in Supporting Information). The conversion efficiency ranged between 11% and 26%. In Figure 4B, we compare the theoretically predicted temperature increase from eq 2 with the experimentally determined via XRD for AuNR 3.9 11%. The corresponding data for AuNR 4.4 11% is included in Supporting Information (Figure S.14). Figure 4B shows that in the linear temperature-power region, the predicted temperature increase based on eq 2 aligns well with the experimentally determined when using the absorption cross section calculated based on the AuNR dimensions. However, when using the cross sections determined via extinction spectroscopy, the predicted temperature increase is greater than the experimentally determined. This is likely due to the scattering contribution to the extinction cross section being greater than what was predicted with the theoretical calculations. As the theoretical predictions assume that the properties of the systems are temperature-independent, we naturally observe greater deviations from the XRD data at the onset of morphology transformations in the AuNRs. The observed discrepancies between the theoretical predictions and the XRD data highlight the importance of experimentally evaluating the photothermal properties of systems like the ones studied here. The use of a size distribution of randomly arranged particles that exhibit temperature-induced morphology transformations creates a complexity that is overlooked by the assumptions made in the theoretical predictions.

We observed no significant differences between the two populations of AuNRs studied (aspect ratio 3.9 and 4.4) regarding the temperature reached for a set power as determined with XRD. However, the theoretical predictions deviate from the experimental findings to a greater extent for AuNR 4.4 11% compared to AuNR 3.9 11% (Figure S.14). We attribute this to the different levels of clustering in the samples, wherein the AuNR 4.4 11% contains more clusters and thus can be expected to show larger deviations from the model, which is based on periodic arrays of individual nanoparticles that are not optically coupled.

In summary, we have investigated the photothermal properties of supported AuNRs by monitoring the lattice expansion with *in situ* XRD. We corroborated our *in situ* data sets with SEM and Vis-NIR spectroscopy characterization, and compared the experimental findings with theoretical predictions. Based on the results, we could establish a correlation between the laser power, temperature of the AuNRs, and the onset of morphological transformations (Figure 4A). The findings provide fundamental understanding of these systems, giving valuable insights for their practical implementation in PTT applications. The onset of morphological transformations in the supported AuNRs around 120 °C sets a threshold on what temperatures can be employed for therapeutic purposes without inducing irreversible changes in the material properties. Our study further shows that by adjusting the surface coverage of AuNRs, it is possible to tune the laser power required to reach this temperature threshold. Within the accuracy of the used methods, we could not observe any significant differences in the photothermal properties of the two studied AuNR populations, or in how the samples responded to continuous NIR irradiation versus conventional furnace heating. Up to 250 °C, the heating-induced morphological transformation of the AuNRs did not affect the orientation of crystallites, as seen in postexperiment texture analysis.

We can thus construct a detailed picture of the temperature evolution of the studied supported AuNRs and propose general trends in this type of functional layer. The referenced theoretical model for regular NP arrays includes several parameters that can be tuned to achieve a desired temperature-power profile. However, we see that the behavior of application-relevant imperfect systems, as the ones studied here where the particle arrangement is more realistic, requires additional considerations. The increased complexity of studying systems composed of a size distribution of randomly arranged particles with temperature-sensitive morphologies necessitates experimental evaluation of their photothermal properties, for which the theoretical predictions miscalculate the obtained temperature for a set laser power.

From our *in situ* XRD data we observed that, in accordance with the theoretical model, the temperature of the AuNRs increases linearly with laser power, however only within a certain temperature range. The achievable temperature for a given type of AuNR and laser power is a function of surface coverage. At 120 °C, well below the melting temperature of bulk gold, we observed an onset of morphological changes in the supported AuNRs. From SEM imaging of postexperiment samples, we can conclude that these changes include (i) a decrease in the aspect ratio of individual nanorods, and (ii) coalescence of clustered nanorods. The transformations cause a shift of the longitudinal absorption peak, which in turn leads to a departure from linear temperature–power profile. Altogether, the findings give valuable insights into the photothermal behavior of supported AuNRs and emphasize ways of tuning these properties, critical for their implementation in PTT applications.

## Supporting Information Available

Experimental details (materials and methods), additional XRD data (texture analysis, raw data from furnace and NIR laser experiments), postexperiment sample characterization (SEM, Vis-NIR), extinction cross section calculations, estimations of photothermal conversion efficiency, extinction spectroscopy measurements, and calculations on heating of plasmonic nanoparticle arrays (PDF)

## Abbreviations

AuNR: gold nanorod
NIR: near-infrared
XRD: X-ray diffraction
