# Supplementary material for: Photothermal Properties of Solid-Supported Gold Nanorods
Source: Nano Lett. 2024 Sep 30;24(40):12529–35. doi: 10.1021/acs.nanolett.4c03472 (PMC11468669; doi:10.1021/acs.nanolett.4c03472)
Supplement: Supplementary file 1 — nl4c03472_si_001.pdf [file nl4c03472_si_001.pdf]

Supporting information

Photothermal properties of solid-supported gold nanorods

Maja Uusitalo[†,§], *Michal Strach*[‡,§], *Gustav Eriksson*[†], *Tetiana Dmytrenko*[‡], *John Andersson*[†], *Andreas Dahlin*[†], *Mats Hulander*[†], *Martin Andersson*[†,]*

† Department of Chemistry and Chemical Engineering, Chalmers University of Technology, SE-412 96 Gothenburg, Sweden.

‡ Chalmers Materials Analysis Laboratory, Chalmers University of Technology, SE-412 96 Gothenburg, Sweden.

* Corresponding author at: Department of Chemistry and Chemical Engineering, Chalmers University of Technology, SE-412 96 Gothenburg, Sweden

E-mail address: martin.andersson@chalmers.se (M. Andersson)

1. Materials and methods

If not specified otherwise, all chemicals were acquired from Sigma-Aldrich Sweden AB and used as-received. Glassware used for the gold nanorod syntheses and for immobilizing the gold nanorods on glass substrates were cleaned with a basic piranha solution. The basic piranha solution was prepared by mixing ultrapure water (Milli-Q®, 18.2 MΩ·cm, Merck Millipore) with ammonia solution (28%, VWR) and hydrogen peroxide (30%, Fisher Scientific) in a 5:1:1 ratio and heating it to 70 °C. Once cleaning was completed, the glassware was thoroughly rinsed with ultrapure water and dried with nitrogen gas. The near-infrared laser used has a central wavelength of 808 ± 3 nm and was purchased from BWT Beijing Ltd. (model DS3-51523-50.00W). The optical fiber of the laser was connected to an air-spaced doublet collimator (F810SMA-780, Thorlabs), which in turn was attached to a 2X beam expander (GBE02-B, Thorlabs), yielding a beam diameter of 19 mm. The irradiance output of the laser system was measured using an optical power meter (Thorlabs, PM160T-HP), data are shown in Fig. S. 1 and Table S. 1.

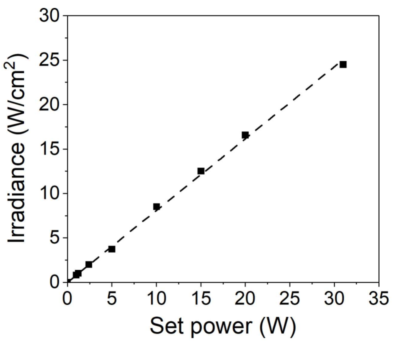

Fig. S. 1. Irradiance output for different set powers from the NIR laser (808 nm) with collimator and beam expander attached. The irradiance was measured with an optical power meter (Thorlabs, PM160T-HP). Linear fit y = 0.80856, R^2 = 0.9992, intercept set to (0,0).

Table S. 1. Irradiance output for the set powers used in the NIR laser heating experiments. Irradiance output determined from the linear fit in Fig. S. 1.

Power (W)	Irradiance (W/cm^2)
2	1.6
4	3.2
6	4.9
8	6.5
10	8.1
12	9.7
14	11.3
16	12.9
20	16.2
24	19.4

1.1 Gold nanorod syntheses and purification

The gold nanorod synthesis protocols were adapted from a seed-mediated procedure described elsewhere,[1] wherein the concentration of silver nitrate ($AgNO_3$) and ascorbic acid was varied to tune the gold nanorods' dimensions. Two types of gold nanorods were synthesised, one with an average aspect ratio of 3.9 (AuNR 3.9) and the other with an average aspect ratio of 4.4 (AuNR 4.4). In short, a seed suspension was prepared in a 30 °C water bath by adding 25 µl of 50 mM gold(III) chloride ($HAuCl_4$) solution to 4.7 ml of 100 mM hexadecyltrimethylammonium bromide (CTAB) solution. Afterward, 300 µl of 10 mM sodium borohydride was added under strong stirring. The resulting seed suspension was kept mildly stirring at 30 °C until use.

For the gold nanorods with an aspect ratio of 3.9, a growth solution was prepared in 30 °C water bath by adding 1.14 ml of 1 M HCl and 600 µl of 50 mM $HAuCl_4$ to 60 ml of 100 mM CTAB solution. Thereafter, 720 µl of 10 mM $AgNO_3$ was added, followed by 600 µl of 100 mM ascorbic acid. Lastly, 144 µl of the seed suspension was added to the growth solution, which was thoroughly mixed and then left undisturbed at 30 °C for 1 h and 50 min. For the gold nanorods with an aspect ratio of 4.4, the growth solution was prepared identically, but the $AgNO_3$ concentration was increased to 11 mM and the ascorbic acid concentration lowered to 90 mM. After addition of the 144 µl of seed suspension, the mixture was left undisturbed at 30°C for 2 h. The synthesised gold nanorods were purified from their reaction mixtures by centrifugation with redispersion in ultrapure water.

For AuNR 3.9, the entire synthesis suspension was centrifuged at 1900 xg for 30 min at 28 °C, whereafter the pellet with gold nanorods was collected and redispersed in ultrapure water. The suspension was centrifuged a second time at 1800 xg for 35 min at 28 °C before the pellet was redispersed in ultrapure water. The suspension was centrifuged a third time at 1800 xg for 35 min at 28 °C, after which the gold nanorod pellet was redispersed in ultrapure water a final time, generating the stock AuNR 3.9 suspension.

For AuNR 4.4, the entire synthesis suspension was centrifuged at 1800 xg for 45 min at 28 °C before the gold nanorod pellet was redispersed in ultrapure water. The centrifugation and redispersion was performed two additional times, generating the stock AuNR 4.4 suspension.

The final concentrated gold nanorod suspensions were used as stocks for the surface-immobilization procedures. The AuNR suspensions were stored at 4 °C protected from light.

1.2 Immobilization of gold nanorods on glass

The synthesised gold nanorods were immobilized onto glass cover slips (13 mm diameter, #1.5, VWR) through electrostatic interaction between the cationic nanorods and the anionic glass surface. The glass substrates were rinsed with 95% ethanol (Solveco AB) followed by ultrapure water before being dried with N_2. The substrates were thereafter placed in a glass petri dish and nitric acid (65-67%) was added to cover. The nitric acid immersion was conducted overnight (18-19 h), whereafter the pre-treated glass substrates were thoroughly rinsed with ultrapure water before being immersed in AuNR suspension. To tune the surface coverage, the immersion time and the concentration of the AuNR suspension were varied. AuNR suspensions were prepared from the stocks by diluting them with ultrapure water, and the gold concentration was estimated from the absorbance at 400 nm using a correlation described previously.[1] The relation describes that an absorption at 400 nm of 1.2 corresponds to $[Au^0] = 0.5$ mM. Based on this, $A_{400\ nm} = 0.3 \Leftrightarrow [Au^0] = 0.125$ mM = 24.6 μg/ml. The 11% surface coverage samples were immersed in 25 μg/ml AuNR suspensions (of AuNR 3.9 and AuNR 4.4, respectively) for 4 h. For the 13% surface coverage sample, the AuNR 3.9 suspension had a concentration of 30 μg/ml, and the immersion time was extended to 5 h. After immersion in gold nanorod suspension, the suspension was exchanged to ultrapure water before the samples were extracted, immersed in 99.5% ethanol (Solveco AB), and allowed to air-dry.

1.3 *In situ* X-ray diffraction

The main series of furnace heating experiments were carried out on a D8 Advance diffractometer using a Cu X-ray source, PSD and variable slit set to 6 mm sample length. The instrument is equipped with an XRK900 heating chamber from Anton-Paar. As-prepared samples were placed on a Macor ceramic support inside of the chamber. Temperature calibration was carried out using MgO powder deposited on the same glass substrate as used for the AuNRs. Displacement due to thermal dilatation was determined based on a series of Z-scans using the same temperature ramp. AuNR data was corrected accordingly, for the sample displacement error and temperature readout error. We followed the evolution of the (200) Au peak with 10-20 min scans in the 2Theta range 42-46° (scan time depended on the signal-to-noise ratio). Heating ramps were set to 0.1°/s, and the samples were equilibrated for 30 min at each set temperature before the measurement.

We also carried out supporting series of measurements on a SaxsLab MAT:Nordic system, equipped with a microfocus Cu source and Pilatus 300K (SAXS) and 100K (WAXS) detectors from Dectris. This configuration provides lower peak position accuracy but better signal-to-noise ratio due to the focusing X-ray source and large 2D detector, and higher temperature accuracy. Heating was achieved using a Linkam HFSX350 heating stage, and the temperature was calibrated using MgO powder. The experiments were performed with the entire beam path under vacuum. The entire heating stage was mounted at a 22° angle to the incident beam to achieve the Bragg condition for the (200) peak. The sample to detector distance was calibrated using LaB_6 powder, and the data was integrated using Saxsgui software with the necessary corrections. In this case the significant thermal dilatation of the stage, which could not be precisely corrected for due to the geometry of the setup, prevented us from lattice parameter determination. Hence, these results were only used to observe changes in the peak shapes as a function of temperature. Due to significant instrumental broadening of the peaks, for these datasets we follow the increase in the intensity rather than Full Width Half Maximum (FWHM).

Texture analysis experiments were performed on a Bruker D8 Discover equipped with a Cu X-ray source, a compact Eulerian cradle (Phi, Z, Chi), and Eiger 2D photon counting detector. Samples were aligned in the beam, and patterns were collected with varying Phi and Chi, up to 80°.

The NIR laser heating experiments were carried out on the same D8 Discover diffractometer with the laser placed inside of the enclosure of the instrument. The samples were placed on two 0.5 mm OD borosilicate glass capillaries with a wall thickness of 0.01 mm and aligned with the X-ray beam. The laser system's collimator was placed 10 cm away from the sample at a 90° angle, ensuring that the entire sample surface was illuminated, and the flux was comparable between experiments. The support stage was designed to ensure the NIR beam was not heating any elements within the vicinity of the sample, except the AuNRs. We monitored the peak evolution at each power plateau until no more changes were observed in the obtained patterns (usually within 10-30 min, depending on laser power). Scan time was set to allow for a satisfactory signal-to-noise ratio, and was usually 5-10 min. Control experiments were performed between the irradiation steps to follow the evolution of room temperature peak shapes at each stage. Collected data was analyzed using EVA, TOPAS, and TEXTURE software from Bruker. Background was simulated with a Chebychev polynomial with between 1 and 3 coefficients. Peaks were chosen to be fitted with a PearsonVII or Split PearsonVII functions in TOPAS.

2. Transmission electron microscopy (TEM) characterization

Transmission electron microscopy (TEM) analysis was conducted using a FEI Titan 80-300 operated in TEM mode at 300 kV. TEM samples were prepared by placing 5 µl AuNR suspension on UV/O_3-treated copper grids with an ultrathin carbon film on a lacey carbon support film (TED Pella). The AuNR suspensions were prepared from the stock suspensions by diluting them with ultrapure water to an estimated gold concentration of 25 µg/ml. Excess AuNR suspension was removed by blotting the grid with filter paper. The obtained TEM micrographs were analyzed in Gatan Microscopy Suite 3. The AuNR dimensions (Table S. 2, Fig. S. 2) were determined from TEM micrographs by measuring 400 particles of each morphology using the image analysis software Fiji (ImageJ).

Table S. 2. AuNR dimensions expressed as average ± standard deviation, determined from measuring 400 particles in TEM micrographs. For each particle the length, the width in the middle and the width of each end were measured. The values for width ends are calculated from the average end width of each AuNR, and the values for width overall are calculated from the average of all three widths measured of each AuNR. Aspect ratio = length/width overall.

	Length (nm)	Width middle (nm)	Width ends (nm)	Width overall (nm)	Aspect ratio
AuNR 3.9	67.2 ± 6.9	16.1 ± 2.0	18.4 ± 2.2	17.6 ± 2.1	3.9 ± 0.5
AuNR 4.4	66.0 ± 9.7	14.7 ± 2.2	15.3 ± 2.1	15.1 ± 2.1	4.4 ± 0.7

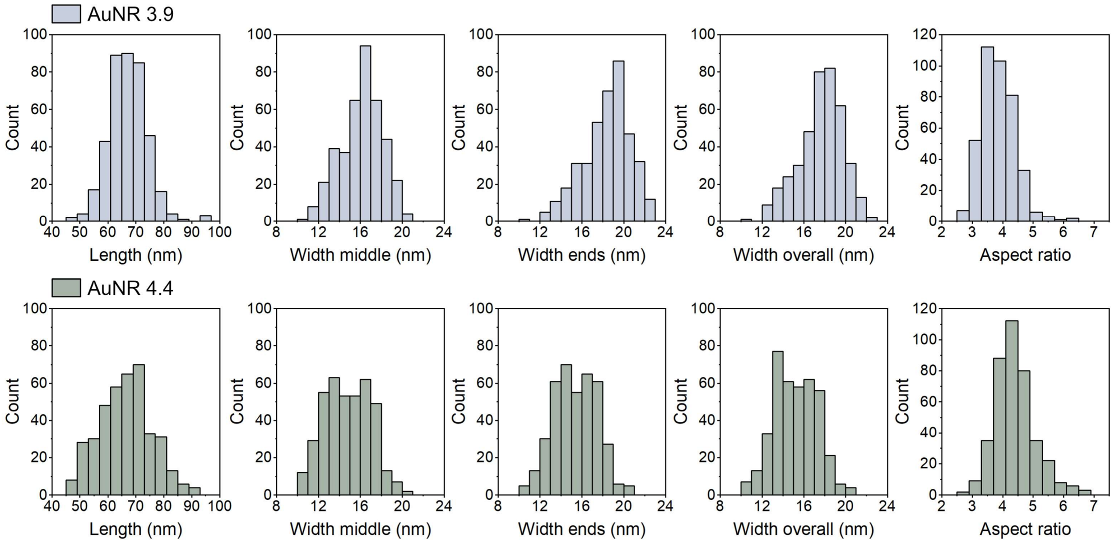

Fig. S. 2. Histograms of the distribution of the gold nanorods' dimensions.

3. Scanning electron microscopy (SEM) characterization

The AuNR surface-immobilization was characterized using a Zeiss Ultra 55 FEG scanning electron microscope. The samples of AuNRs supported on glass were imaged in secondary electron mode using the SE2 detector and acceleration voltages between 2-3 kV. The SEM micrographs were analyzed with respect to the surface coverage of gold nanorods (projected area covered by nanoparticles). For the determination of the surface coverage, 12 micrographs of each sample type were analyzed.

4. XRD raw patterns, texture analysis, furnace heating, NIR experiments

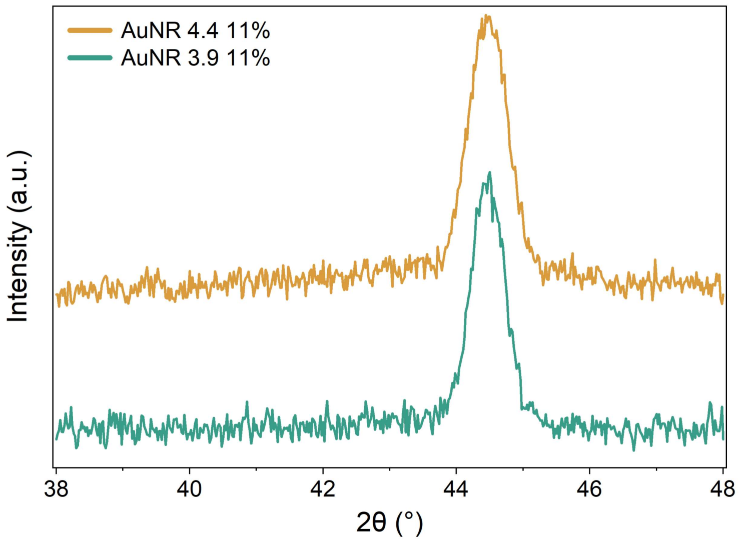

Fig. S. 3. XRD patterns of raw, untreated AuNR 3.9 11% and AuNR 4.4 11% samples.

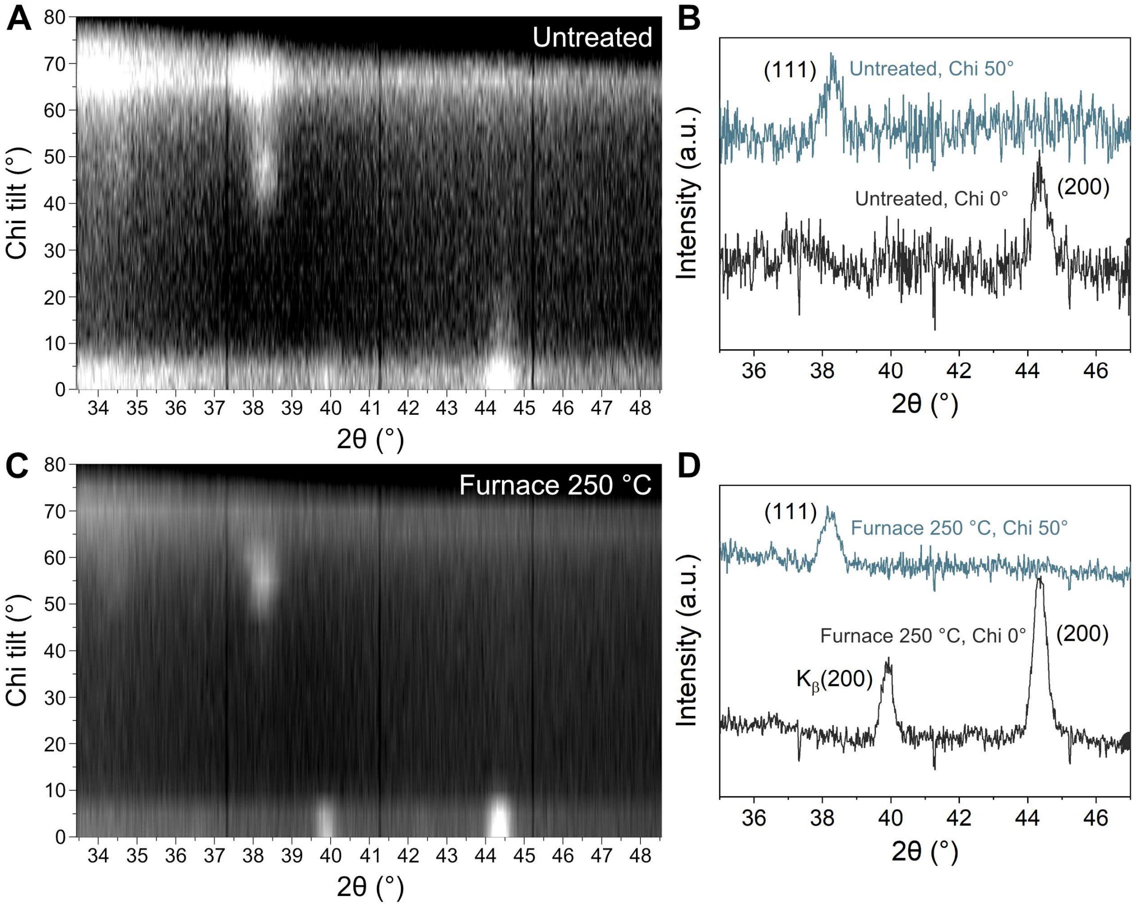

Fig. S. 4. Intensity maps from the texture analysis experiment for a (A) untreated, and (C) furnace heated AuNR 3.9 11% samples. (B) and (D) XRD patterns showing the (200) peak at Chi = 0° and (111) peak at Chi = 50° for the respective samples.

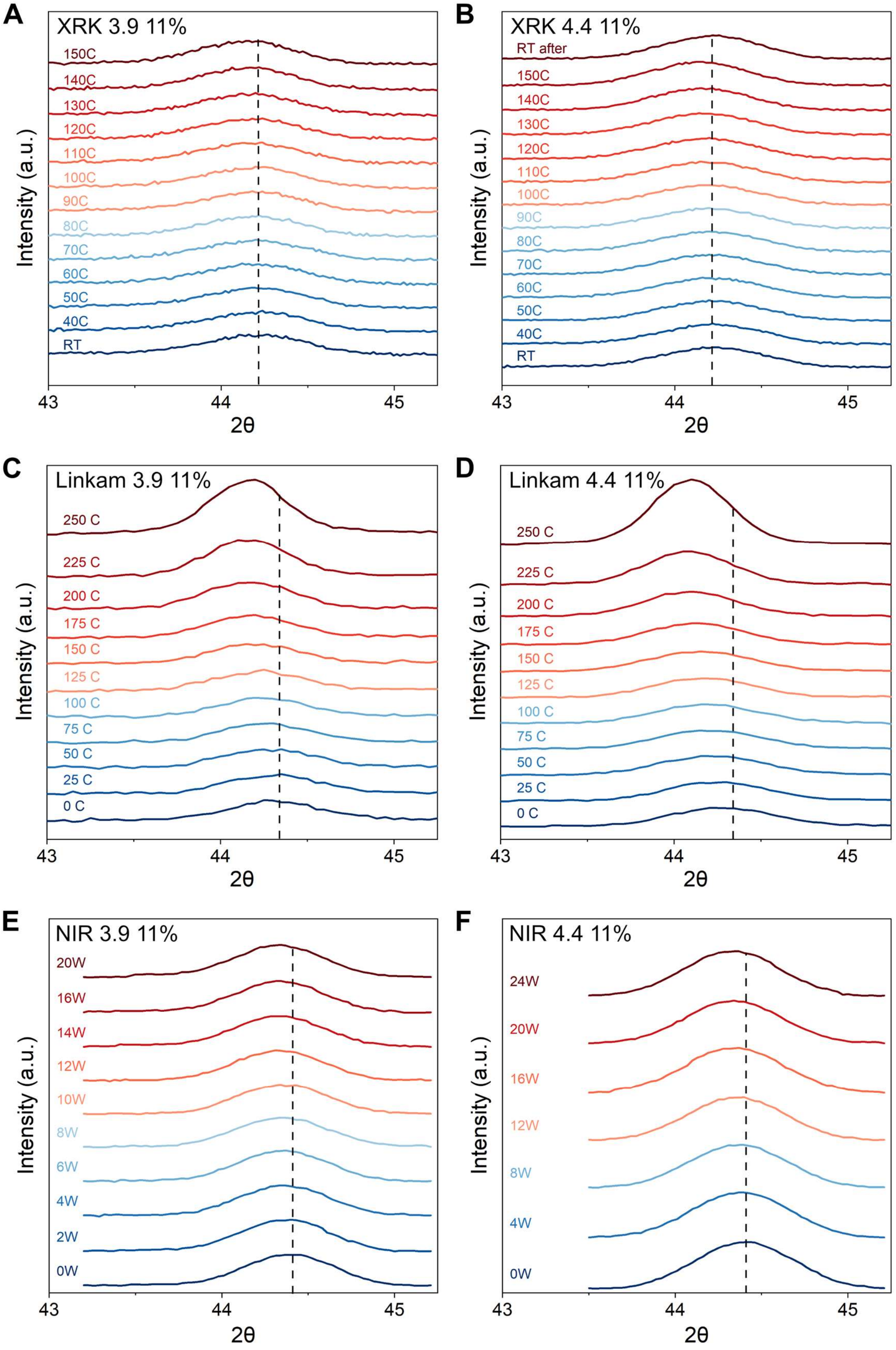

Fig. S. 5. Raw diffraction patterns from the *in situ* XRD experiments for AuNR 3.9 11% (left) and AuNR 4.4 11% (right). (A) and (B) XRK heating up to 150 °C, (C) and (D) Linkam stage heating up to 250 °C, and (E) and (F) NIR laser heating. The differences in the initial peak position at ambient conditions are due to the use of different sample stages. Lattice parameters were corrected accordingly.

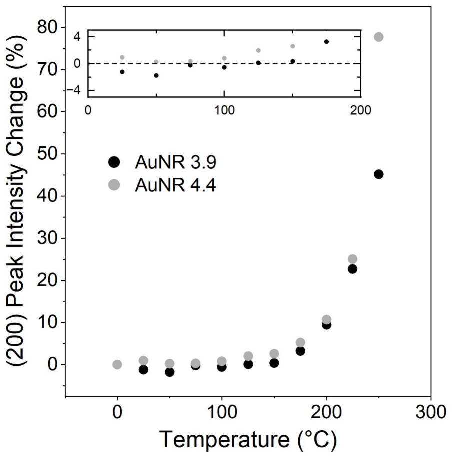

Fig. S. 6. The evolution of the (200) peak intensity as a function of temperature during heating up to 250 °C on the Linkam stage for the AuNR 3.9 11% and AuNR 4.4 11%. Inset shows a close-up of the initial increase up to 5%.

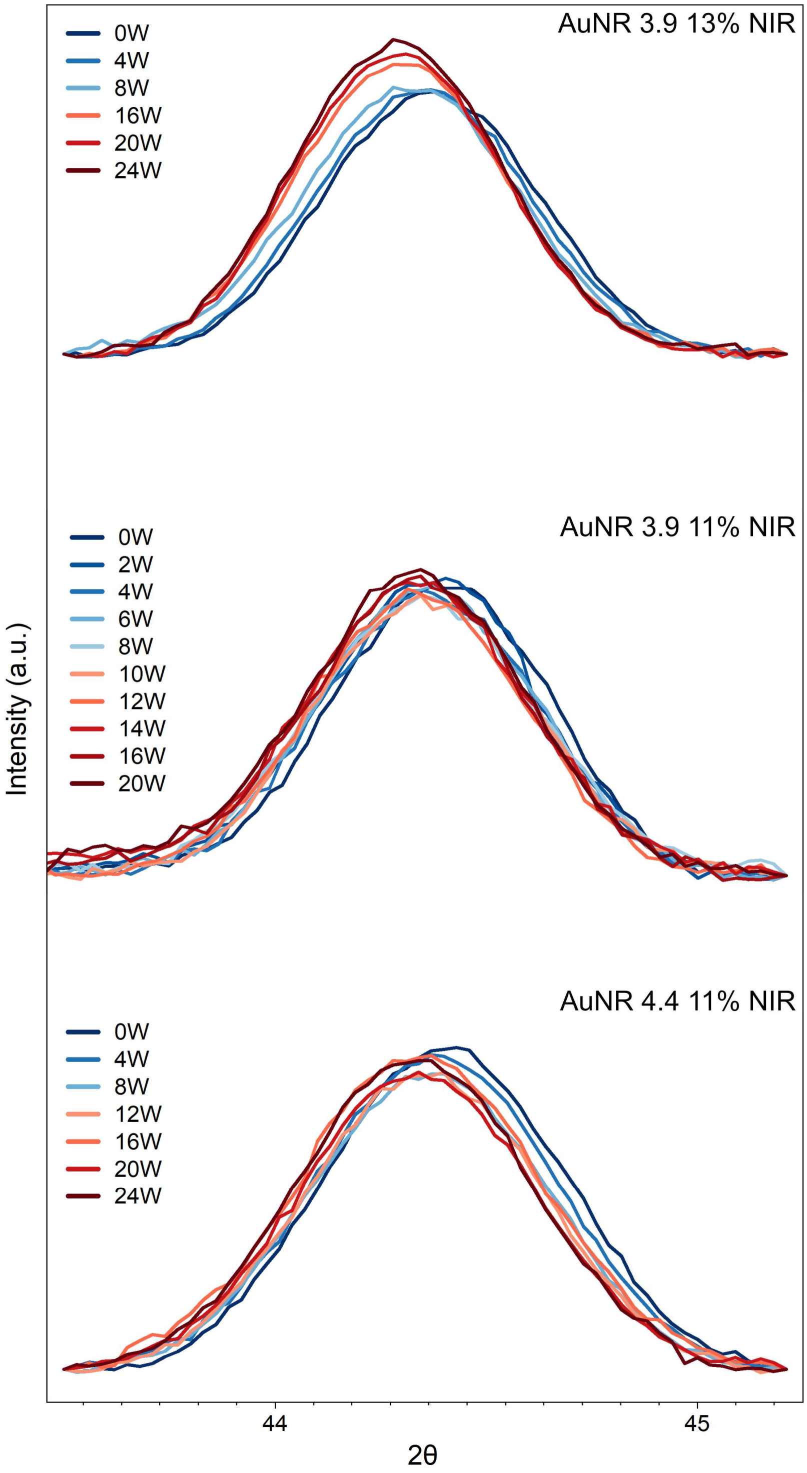

Fig. S. 7. Diffraction patterns from the *in situ* XRD NIR laser heating experiments for AuNR 3.9 13% (top), AuNR 3.9 11% (middle), and AuNR 4.4 11% (bottom).

In the AuNR 3.9 13% sample there is a clear modification of the peak shape during NIR heating (Fig. S. 7), like what was observed during conventional furnace heating (Fig. S. 5). Interestingly, for the samples with 11% coverage, the FWHM or peak intensity did not change significantly during NIR heating regardless of the laser power, although the peak shifts corresponded to the reported temperature increase above 120 °C. The only indications of morphological changes in the 11% coverage samples are the departure from a linear temperature increase with power and the post-experiment Vis-NIR spectra (Figure 5, Fig. S. 11). This disparity might be connected to the lower temperatures reached for the 11% coverage samples compared to the 13% for the same laser power values. Apparently, the absorption spectra are more sensitive to the onset of the morphological changes than the diffraction peak characteristics.

5. Characterization of post-experiment samples

Scanning electron microscopy (SEM)

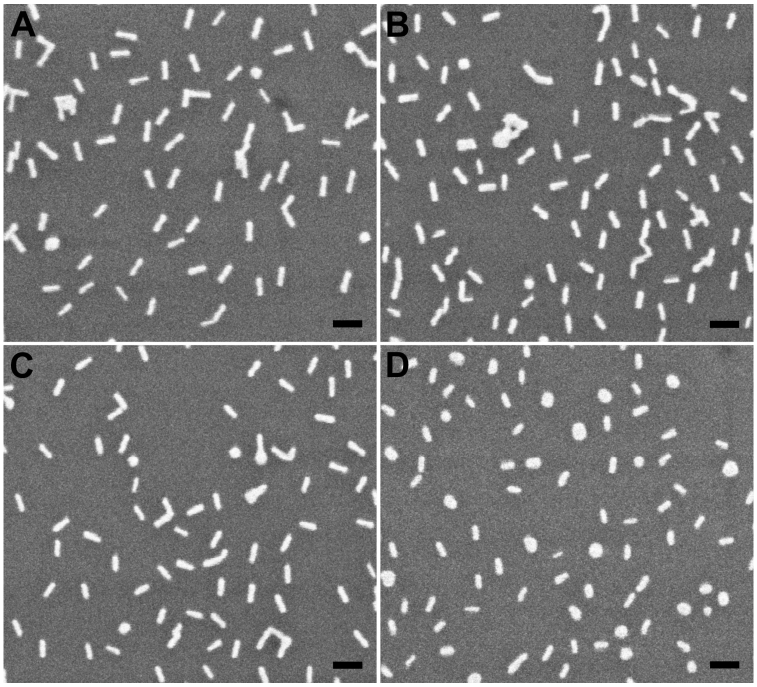

Fig. S. 8. SEM micrographs of AuNR 3.9 11%, untreated and after NIR laser irradiation or furnace heating. (A) Untreated, (B) NIR irradiation up to 20 W, (C) furnace heating to 150 °C, and (D) furnace heating to 250 °C. Scale bars are 100 nm.

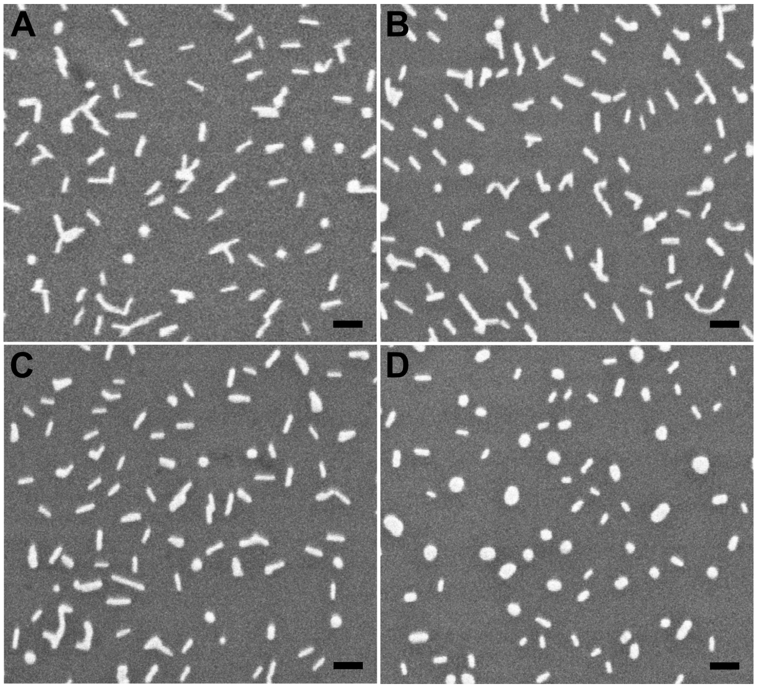

Fig. S. 9. SEM micrographs of AuNR 4.4 11%, untreated and after NIR laser irradiation or furnace heating. (A) Untreated, (B) NIR irradiation up to 24 W, (C) furnace heating to 150 °C, and (D) furnace heating to 250 °C. Scale bars are 100 nm.

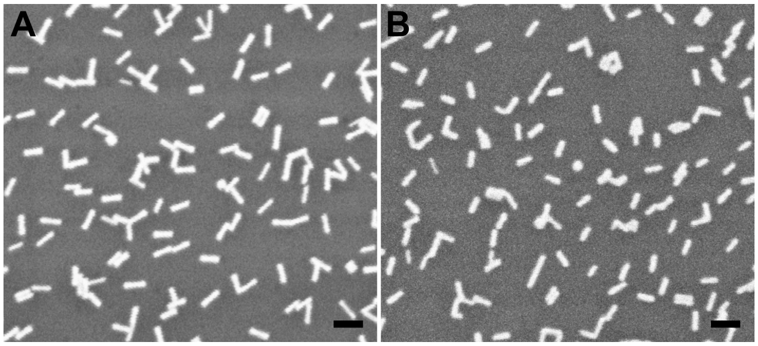

Fig. S. 10. SEM micrographs of AuNR 3.9 13%, untreated and after NIR laser irradiation. (A) Untreated, and (B) NIR irradiation up to 24 W. Scale bars are 100 nm.

Vis-NIR spectroscopy

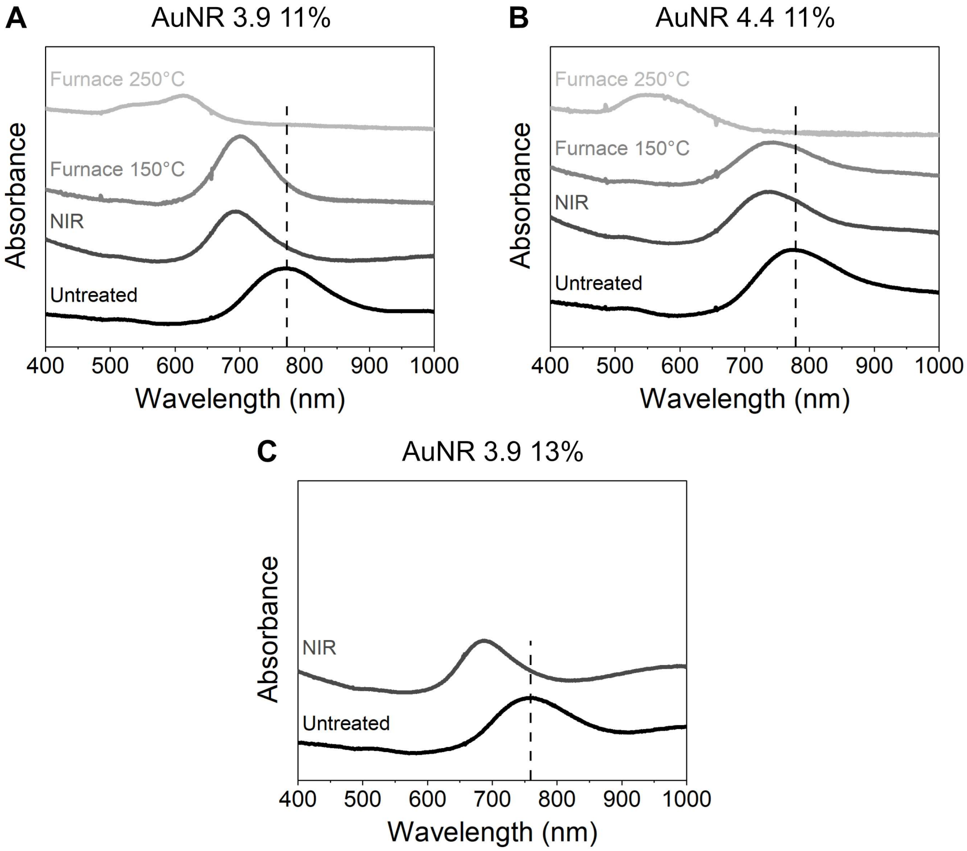

Fig. S. 11. Vis-NIR spectra of untreated samples, samples after NIR laser heating, and conventional furnace heating to 150 °C and 250 °C. (A) AuNR 3.9 11% (NIR up to 20 W), (B) AuNR 4.4 11% (NIR up to 24 W), and (C) AuNR 3.9 13% (NIR up to 24 W).

6. Theory: calculations of extinction, scattering, and absorption cross section

For both aspect ratios of AuNRs (3.9 and 4.4), the extinction, scattering, and absorption cross sections were calculated with the following methodology.

The extinction cross section can be obtained from[2]

$$\sigma_{ext} = kIm(\alpha)$$

Where $k = 2\pi/\lambda$, the wavenumber, and α the polarizability.

The scattering cross section was determined from[3,4]

$$\sigma_{sca} = \frac{k^4}{6\pi}|\alpha|^2$$

And thereafter the absorption cross section could be obtained as[2]

$$\sigma_{abs} = \sigma_{ext} - \sigma_{sca}$$

The polarizability of an ellipsoid can be determined from[5]

$$\alpha = V\frac{\varepsilon-\varepsilon_m}{\varepsilon_m+L[\varepsilon-\varepsilon_m]}$$

Where V is the nanoparticle volume, ε is the dielectric function of the material (gold at 808 nm, taken from literature)[6], ε_m is the dielectric function of the medium (average of glass and air), and L the shape factor.

The volume of an ellipsoid is

$$V = \frac{4\pi R_1R_2R_3}{3}$$

Which for a prolate spheroid (nanorod) becomes

$$V = \frac{4\pi R_1^2R_3}{3}$$

As $R_3 > R_1 = R_2$. For the nanorods under study, R_3 is half the length, and R_1 half the width.

As the light will be polarized both parallel and perpendicular to the nanorod axis, the cross sections (σ_{ext}, σ_{sca}, σ_{abs}) were determined as averages of the cross sections calculated with parallel polarizability, $\alpha_{\parallel}$, and the perpendicular polarizability, $\alpha_{\perp}$.

$$\alpha_{\parallel} = V\frac{\varepsilon-\varepsilon_m}{\varepsilon_m+L_{\parallel}[\varepsilon-\varepsilon_m]}$$

$$\alpha_{\perp} = V\frac{\varepsilon-\varepsilon_m}{\varepsilon_m+L_{\perp}[\varepsilon-\varepsilon_m]}$$

The shape factor of a prolate spheroid [5] (nanorod) for polarization parallel to the axis was determined from

$$L_{\parallel} = \frac{\left[\frac{R_1}{R_3}\right]^2}{1-\left[\frac{R_1}{R_3}\right]^2}\left[\frac{1}{2\sqrt{1-\left[\frac{R_1}{R_3}\right]^2}}log\left(\frac{1+\sqrt{1-\left[\frac{R_1}{R_3}\right]^2}}{1-\sqrt{1-\left[\frac{R_1}{R_3}\right]^2}}\right)-1\right]$$

And the shape factor for polarization perpendicular to the nanorod axis from

$$L_{\perp} = \frac{\left[\frac{R_3}{R_1}\right]^2}{1-\left[\frac{R_3}{R_1}\right]^2}\left[\frac{1}{2\sqrt{1-\left[\frac{R_3}{R_1}\right]^2}}log\left(\frac{1+\sqrt{1-\left[\frac{R_3}{R_1}\right]^2}}{1-\sqrt{1-\left[\frac{R_3}{R_1}\right]^2}}\right)-1\right]$$

For both aspect ratios of AuNRs, a MATLAB script was used to calculate σ_{ext}, σ_{sca} and σ_{abs} for all 400 particle dimensions (values of R_1 and R_3) determined from TEM micrographs. Table S. 3 shows the average, minimum, and maximum values from these calculations. As the cross sections at a specific wavelength are strongly dependent on the AuNR dimensions, it is expected that they will vary greatly over a size distribution of particles, as can be observed in Table S. 3.

Table S. 3. Extinction, scattering, and absorption cross sections at 808 nm calculated based on the dimensions of the AuNRs. Average, minimum, and maximum values from calculating the cross sections for all 400 AuNRs measured during the size determination from TEM micrographs.

	**AuNR 3.9**			**AuNR 4.4**		
	**Avg**	Min	Max	**Avg**	Min	Max
$\sigma_{ext}\ (nm^2)$	**1398**	59	14907	**2710**	31	17952
$\sigma_{sca}\ (nm^2)$	**197**	7	2708	**338**	2	3681
$\sigma_{abs}\ (nm^2)$	**1201**	50	12199	**2373**	28	14395
$\sigma_{abs}\ /\sigma_{ext}$	0.844	-	-	0.885	-	-

7. Photothermal conversion efficiency

The photothermal conversion efficiencies of the samples were estimated from the surface coverage and the calculated absorption cross sections (Table S. 3) of the AuNRs. The surface coverage was converted from area% to #AuNR/area using the average dimensions of the AuNRs.

$$\#AuNR/m^2 = \frac{area\%\ /\ 100}{l_{AuNR} \times w_{AuNR}}$$

Were l_{AuNR} and w_{AuNR} is the AuNR average length and width (in meters), respectively. The conversion efficiencies were estimated by multiplying the surface coverage (#AuNR/area) with the absorption cross sections (area) of the particles, and thus represent the fraction of the incident power contributing to the heat generation upon irradiation with the NIR laser. Results are presented in Table S. 4.

Table S. 4. Estimated photothermal conversion efficiency of the supported AuNR samples.

	**AuNR 3.9 11%**	**AuNR 3.9 13%**	**AuNR 4.4 11%**
Surface coverage (%)	10.7	13.4	11.0
Surface coverage ($AuNR/m^2$)	9.05×10^{13}	1.13×10^{14}	1.10×10^{14}
$\sigma_{abs}\ (m^2)$[1]	1.20×10^{-15}	1.20×10^{-15}	2.37×10^{-15}
Conversion efficiency ($surface\ coverage\ \times\ \sigma_{abs}$)	0.11	0.14	0.26

[1] Absorption cross section calculated based on the AuNRs' dimensions (Table S. 3)

8. Extinction spectroscopy: experimental determination of extinction cross sections and evaluation of temperature dependence of surface plasmon resonance

Experimental determination of extinction cross section

The extinction spectra were measured on a home-built microscopy/transmission setup equipped with a halogen lamp (Newport) and a 10x air objective (NA 0.25) illuminating the sample. The transmitted light was collected with a photodiode array spectrometer (B&WTEK). Dark counts (with the light source turned off) were subtracted from the measured intensities, and the reference intensity was measured through a bare glass substrate. Extinction spectra for AuNR 3.9 11% and AuNR 4.4 11% are shown in Fig. S. 12.

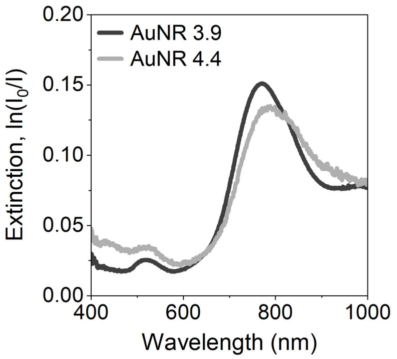

Fig. S. 12. Extinction spectra of AuNR 3.9 11% and AuNR 4.4 11%.

The measured extinction spectra were used to determine the extinction cross section of the AuNRs. The average values of the extinction, *ln(I₀/I)*, at 808 nm from measuring spectra at four different locations on the samples were determined. The extinction cross sections were calculated from the following expression:

$$\sigma_{ext} = \frac{A}{N_{AuNR}}\left(1 - \frac{I}{I_0}\right) \qquad \text{(Eq. S.1)}$$

Where $\frac{N_{AuNR}}{A}$, the number of AuNRs per area, was determined through image analysis of SEM micrographs (n = 11).

For each AuNR population, the extinction cross section was determined in two ways:

1. Assuming only the individual AuNRs contribute to the extinction at 808 nm, and thereby excluding the clusters when determining $\frac{N_{AuNR}}{A}$.
2. Assuming all entities (individual AuNRs + clusters) contribute to the extinction at 808 nm, and thereby including the clusters when determining $\frac{N_{AuNR}}{A}$ (one cluster = one particle).

The results from the experimental determination of the extinction cross sections are presented in Table S. 5.

Table S. 5. Results from the extinction spectroscopy and the experimental determination of extinction cross sections.

	**AuNR 3.9**		**AuNR 4.4**	
$ln(I_0/I)$	0.134		0.118	
	Individual AuNRs	Individual AuNRs + clusters	Individual AuNRs	Individual AuNRs + clusters
$\frac{N_{AuNR}}{A}$ $(no./\mu m^2)$	39	48	30	48
σ_{ext} (nm^2)[2]	3246	2598	3677	2310
σ_{abs} (nm^2)[3]	2741	2193	3254	2045

Temperature dependence of plasmon resonance

The plasmon resonance in gold nanoparticles has a known temperature dependence, stemming mainly from changes in the permittivity of the metal when the surrounding materials are glass and air.[7,8] To evaluate whether a temperature-induced resonance shift could impact the temperature-power profiles of the supported AuNRs before the onset of morphology transformations around 120 °C, the temperature dependence of the surface plasmon resonance (SPR) of AuNR 4.4 11% was evaluated by measuring the extinction spectrum as a function of temperature. A transparent heating plate (MP-100MH, Kitazato) was employed to heat the sample while monitoring the longitudinal SPR peak. The reference intensity was measured through the same heating plate and bare glass substrate.

Extinction spectra measured during brief heating from 25 °C to 105 °C (Fig. S. 13A) showed no evident shift of the SPR peak. We also continuously monitored the SPR peak during temperature cycling, with sustained heating at 110 °C alternated with cooling (Fig. S. 13B). A non-reversible blue-shift was observed during sustained heating at 110 °C, likely due to morphology transformations of the AuNRs or refractive index changes caused by evaporation of the CTAB double layer adsorbed on the AuNRs. A reversible contribution to the peak shift could also be noted, however as it did not correlate in time with the temperature changes (which were faster), we attribute this to noise from the experimental setup. Based on the findings, we conclude that the reversible resonance shifts due to changes in the permittivity of the AuNRs up to 110 °C must be smaller than the experimental uncertainty (< 1 nm) and expect it not to have any significant impact on the heating of the AuNRs in the linear region of the temperature-power profiles as determined with *in situ* XRD.

Our experimental findings can further be validated with theoretical predictions. The mechanism of the intrinsic red-shift of the plasmon resonance in gold nanoparticles with temperature due to thermal expansion is due to changes in metal permittivity.[7] The thermal expansion of the crystal lattice results in a reduced electron density and a change in bulk plasma frequency, given by [7,8]

$$\omega_p(T) = \frac{\omega_p(T_0)}{\sqrt{1+3\beta[T-T_0]}}$$

[2] Calculated from experimental data using Eq. S.1

[3] Estimated using the average values of $\sigma_{abs}/\sigma_{ext}$ in Table S. 3.

Were $\omega_p(T_0) = 1.32 \times 10^{16}$ Hz is the plasmon frequency of gold at room temperature,[9] and β the linear thermal expansion coefficient. For bulk gold, $\beta = 14.2 \times 10^{-6}$ at 293 K,[10] which we have shown agrees well with the thermal expansion of the supported AuNRs studied here. Upon a 100 °C increase in temperature one thus expects a very small peak shift (well below 1 nm) induced by the thermal expansion of the AuNRs. This can be tested by modifying ω_p accordingly and inserting the new permittivity into the model for σ_{ext}, as has been shown previously.[8]

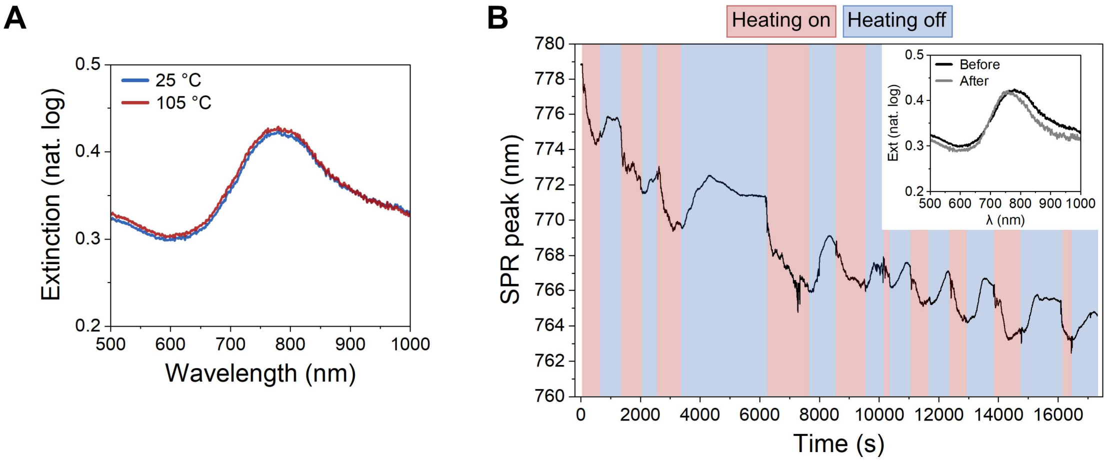

Fig. S. 13. Temperature dependence of surface plasmon resonance for AuNR 4.4 11%. (A) Extinction spectra at 25 and 105 °C, and (B) evolution of SPR peak during temperature cycling, red indicating heating on (110 °C) and blue heating off (cooling occurred under ambient conditions). Inset showing extinction spectra measured at 25 °C before and after the temperature cycling experiment.

9. Theory: heating of plasmonic nanoparticle arrays

For a perfect, regular 2D array of plasmonic nanoparticles under illumination, the relative contribution of the self-temperature increase of a NP (ΔT_0^s) compared to the external temperature increase from the surrounding NPs (ΔT_0^{ext}) can be expressed as a dimensionless parameter ζ_2 (subscript 0 indicating the center of the array).[11]

$$\zeta_2 = \frac{p^2}{3DR}$$

Where p is the periodicity of the NP lattice, D the diameter of the heated area, and R the NP radius (for non-spherical particles an equivalent radius). When ΔT_0^s is dominant ($\zeta_2 >> 1$), the NPs exhibit a temperature increase that is unaffected by the neighboring particles, i.e. the temperature increase is localized around each individual NP. When ΔT_0^{ext} is dominant ($\zeta_2 << 1$), the temperature increase of each NP is primarily caused by heating of the neighboring particles, i.e. collective heating dominates, and the temperature increase is delocalized (homogenous temperature distribution over the entire array). For the AuNR 3.9 11% and AuNR 4.4 11% samples used in this study, ζ_2 was estimated accordingly.

p = 87 nm, determined through image analysis of average nearest neighbor distances in SEM micrographs (n = 12)

D = 13 mm, the glass substrate diameter as the entire sample was illuminated by the laser beam.

$R_{eq} = \left[\frac{(3L-d)d^2}{16}\right]^{1/3}$ for a rod-shaped particle,[12] where L = length and d = width.

$R_{eq}^{AuNR\ .9} = 15.3\ nm,\ R_{eq}^{AuNR\ .4} = 13.8\ nm$

$\zeta_2^{AuNR\ .9} = 1.3 * 10^{-5} \ll 1, \zeta_2^{AuNR\ .4} = 1.4 * 10^{-5} \ll 1$

The systems studied are thus expected to be dominated by collective heating effects, with a homogeneous temperature distribution throughout the entire AuNR array.

To theoretically predict the temperature increase with laser power for the supported AuNRs, we employed the following model. The temperature increase in the center of a 2D infinite square NP array under uniform and circular irradiation can be estimated from:[11]

$$\Delta T_0 = \Delta T_0^S + \Delta T_0^{ext} = \frac{\sigma_{abs} I}{4\pi\bar{\kappa}R} + \frac{\sigma_{abs} ID}{4\bar{\kappa}p^2}\left(1 - \frac{2\sqrt{p^2}}{\sqrt{\pi}D}\right) \quad \text{(Eq. S.2)}$$

Where I is the irradiance, $\bar{\kappa}$ the average thermal conductivity of medium and substrate, D the diameter of the heated area, and p the periodicity of the square lattice. Using Eq. S.2 along with the theoretically calculated absorption cross sections in Table S. 3 and the cross sections determined from extinction spectroscopy in Table S. 5, we can compare the predicted temperature increase with the *in situ* XRD datasets for AuNR 3.9 11% and AuNR 4.4 11% (Fig. S. 14).

The periodicities, p, used in the predictions were determined through image analysis of average nearest neighbor distances in SEM micrographs (n = 12) for both the AuNR 3.9 11% (p = 86.7 ± 6.4 nm) and AuNR 4.4 11% (p = 87.1 ± 8.4 nm). Based on the image analysis, p = 87 nm was used for the predictions using the absorption cross sections determined through calculations based on the AuNR dimensions ("Calculated"), and through extinction spectroscopy measurements assuming both individual and clustered AuNRs contribute to the extinction at 808 nm ("Ext: individual AuNRs + clusters"). For the cross sections determined when assuming only the individual AuNRs contribute to the extinction ("Ext: individual AuNRs"), complementary image analysis was performed to determine the average nearest neighbor distances when clusters were excluded. This gave a periodicity of 96 nm for AuNR 3.9 11% (p = 96.1 ± 4.5 nm), and of 97 nm for AuNR 4.4 11% (p = 96.9 ± 12.8 nm). As the systems studied here consist of randomly arranged particles, using a single value to represent the interparticle distances is a simplification that will influence the accuracy of the theoretically predicted temperature increase. Moreover, an increased surface coverage is accompanied by an increased level of clustering, which means that although the surface coverage of AuNRs increases, this might not be reflected as a decrease in the average nearest neighbor distance (lower value for the periodicity). The lack of direct correlation between the surface coverage and periodicity complicates theoretical predictions of how increasing the surface coverage will influence the temperature-power profiles for the supported AuNRs studied here, and consequently such predictions were deemed beyond the scope of this study.

As shown in Fig. S. 14, the theoretical predictions based on cross sections determined from extinction spectroscopy ("Ext: individual AuNRs" and "Ext: individual AuNRs + clusters") overestimate the temperature increase with laser power for both types of samples. This deviation could be partly due to the scattering contributions to the extinction cross sections of the supported AuNRs being greater than what the theoretical calculations estimate (Table S. 3), which is feasible as clustered particles will scatter more than individual ones. The theoretical predictions based on absorption cross sections calculated from the AuNR dimensions ("Calculated") agree quite well with the experimentally determined temperature increase for the AuNR 3.9 11% in the linear temperature-power region (Fig. S. 14A), but overestimate the temperature for the AuNR 4.4 11%. We attribute this to the higher level of clustering in the AuNR 4.4 11% sample, giving greater deviations from the model which is based on regular arrays of individual NPs.

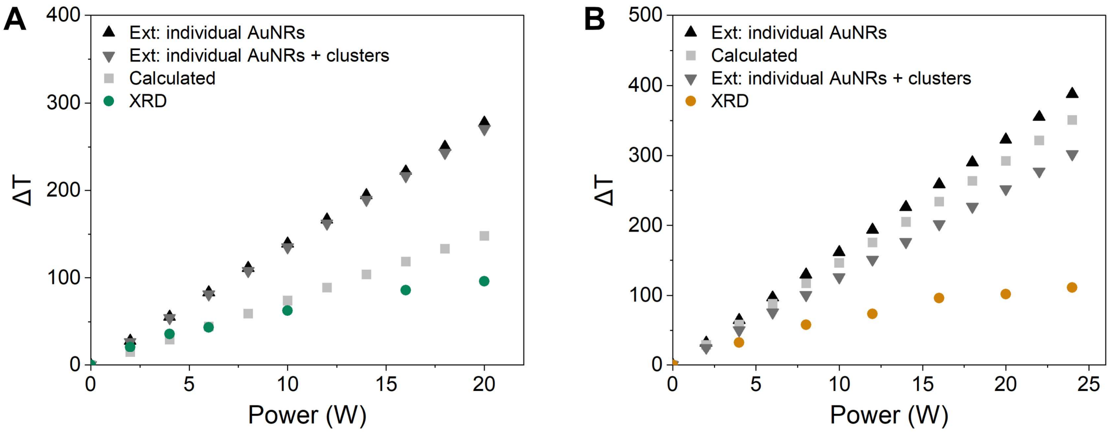

Fig. S. 14. Theoretically predicted (Eq. S.2) and experimentally determined (*in situ* XRD) temperature increase as a function of laser power for (A) AuNR 3.9 11%, and (B) AuNR 4.4 11%. The absorption cross sections used in the theoretical predictions were determined by calculations based on the AuNR dimensions ("Calculated"), or based on extinction spectroscopy measurements assuming either only individual AuNRs ("Ext: individual AuNRs") or both individual and clustered AuNRs ("Ext: individual AuNRs + clusters") contribute to the extinction at 808 nm.

References

(1) Scarabelli, L.; Sánchez-Iglesias, A.; Pérez-Juste, J.; Liz-Marzán, L. M. A "Tips and Tricks" Practical Guide to the Synthesis of Gold Nanorods. *Journal of Physical Chemistry Letters* **2015**, *6* (21), 4270–4279. https://doi.org/10.1021/acs.jpclett.5b02123.

(2) Dahlin, A. B. *Plasmonic Biosensors : An Integrated View of Refractometric Detection.*; IOS Press: Amsterdam, 2012.

(3) Mishchenko, M. I.; Travis, L. D.; Lacis, A. A. *Scattering, Absorption, and Emission of Light by Small Particles*; Cambridge University Press: Cambridge, 2002.

(4) Hulst, H. C. van de. *Light Scattering by Small Particles*; Dover Publications: New York, 1981.

(5) Bohren, C. F.; Huffman, D. R. *Absorption and Scattering of Light by Small Particles*; Wiley: New York, 1998.

(6) Johnson, P. B.; Christy, R. W. Optical Constants of the Noble Metals. *Phys Rev B* **1972**, *6* (12).

(7) Yeshchenko, O. A.; Bondarchuk, I. S.; Gurin, V. S.; Dmitruk, I. M.; Kotko, A. V. Temperature Dependence of the Surface Plasmon Resonance in Gold Nanoparticles. *Surf Sci* **2013**, *608*, 275–281. https://doi.org/10.1016/j.susc.2012.10.019.

(8) Virk, M.; Xiong, K.; Svedendahl, M.; Käll, M.; Dahlin, A. B. A Thermal Plasmonic Sensor Platform: Resistive Heating of Nanohole Arrays. *Nano Lett* **2014**, *14* (6), 3544–3549. https://doi.org/10.1021/nl5011542.

(9) Etchegoin, P. G.; Le Ru, E. C.; Meyer, M. An Analytic Model for the Optical Properties of Gold. *Journal of Chemical Physics* **2006**, *125* (16). https://doi.org/10.1063/1.2360270.

(10) Touloukian, Y. S.; Kirby, Y. B.; Taylor, R. E.; Desai, P. D. Thermal Expansion: Metallic Elements and Alloys. In *Thermophysical Properties of Matter*; IFI/Plenum: New York, 1975; Vol. 12.

(11) Baffou, G.; Berto, P.; Bermúdez Ureña, E.; Quidant, R.; Monneret, S.; Polleux, J.; Rigneault, H. Photoinduced Heating of Nanoparticle Arrays. *ACS Nano* **2013**, *7* (8), 6478–6488. https://doi.org/10.1021/nn401924n.

(12) Baffou, G.; Quidant, R.; García De Abajo, F. J. Nanoscale Control of Optical Heating in Complex Plasmonic Systems. *ACS Nano* **2010**, *4* (2), 709–716. https://doi.org/10.1021/nn901144d.
